# Treatment of Acute Promyelocytic Leukemia with AIDA Based Regimen. Update of a Tunisian Single Center Study

**DOI:** 10.4084/MJHID.2011.033

**Published:** 2011-09-08

**Authors:** Ramzi Jeddi, Hèla Ghédira, Ramzi Ben Amor, Yosr Ben Abdennebi, Kacem Karima, Zarrouk Mohamed, Hend Ben Neji, Lamia Aissaoui, Raihane Ben Lakhal, Naouel Ben Salah, Samia Menif, Zaher Belhadjali, Hela Ben Abid, Emna Gouider, Raouf Hafsia, Ali Saad, Pierre Fenaux, Balkis Meddeb

**Affiliations:** 1 Hematology Department, Aziza Othmana University Hospital, Tunis, Tunisia; 2 Department of molecular analysis, Pasteur Institute, Tunis, Tunisia; 3 Department of cytogenetic analysis, Farhat Hached Hospital, Sousse, Tunisia; 4 Hematology Department, Avicenne Hospital, Assistance Publique Hôpitaux de Paris, Paris 13 University, Paris, France

## Abstract

In Tunisia, the ATRA era began in 1998 with the use, consecutively, of two regimens combining ATRA and an anthracycline with cytarabine (APL93), and without cytarabine (LPA99). From 2004, 51 patients with confirmed APL either by t(15;17) or PML/RARA were treated according to the PETHEMA LPA 99 trial. Forty three patients achieved CR (86%). The remaining seven patients had early death (one died before treatment onset): four caused by differentiation syndrome (DS) and three died from central nervous system hemorrhage. Multivariate analysis revealed that female gender (P=0.045), baseline WBC> 10 G/L (P=0.041) and serum creatinine > 1.4mg/dl (P=0.021) were predictive of mortality during induction. DS was observed in 16 patients (32%) after a median onset time of 15 days from treatment onset (range, 2–29). Body mass index ≥ 30 (P=0.01) remained independent predictor of DS. Occurrence of hypertensive peaks significantly predicted occurrence of DS (P=0.011) and was significantly associated with high BMI (p=0.003). With a median follow-up of 50 months, 5 year cumulative incidence of relapse, event free and overall survival were 4.7%, 74% and 78%, respectively.

## Introduction:

Acute promyelocytic leukemia (APL) has now become the most curable of all subtypes of acute myeloid leukemia. A major advance was the introduction of all-trans retinoic acid (ATRA), which induces differentiation of the leukemic promyelocytes.^1^–^4^ In combination with anthracyclines, cure rates of 75–80% are now reported.^1^–^3^ Two challenges however remain: reducing early mortality, which remains at approximately 10%, and preventing relapses.^1^–^7^ In Tunisia, the ATRA era begun in 1998 with the use, consecutively, of two regimens combining ATRA with an anthracycline and cytarabine (APL93), and an anthracycline without cytarabine (LPA99). Prior to the advent of ATRA we had treated 20 patients with morphologically recognized APL with a combination of cytarabine 200 mg/m^2^ (days 1–7) and daunorubicin 45 mg/m^2^ (days 1–3). The remission induction rate was 35.7%, the early death 42.8%, and overall survival 10% at 6 years (unpublished data).

Between 1998 and 2004, 34 consecutive patients with genetically confirmed APL from two hematology departments (University Hospital of Tunis and Sfax) were treated with the European APL93 trial in which, induction treatment consisted of ATRA, and Daunorubicin associated to cytarabine. The complete remission rate was 82%. Four year event free survival, cumulative incidence of relapse (CIR) and overall survival were 63.4%, 14.2%, 69.7% respectively.^8^

Since 2004, 51 genetically confirmed APL have been treated by the PETHEMA LPA 99 trial avoiding cytarabine during induction and consolidation cycles in order to reduce toxicity without increasing the incidence of relapse.^2^ We already reported our preliminary experience with this protocol in 39 patients with relatively limited follow up.^9^ We update here our results on 51 patients and with longer follow up.

## Methods:

Eligibility criteria in this study were a diagnosis of APL with demonstration of the t(15;17) or PML/RARA rearrangement. Treatment schedule was similar to that of the Spanish PETHEMA LPA99 trial: Induction therapy consisted of oral ATRA 45 mg/m^2^/day until morphological CR and intravenous idarubicin 12 mg/m^2^ on days 2, 4, 6, and 8. For patients aged 20 years or younger, the ATRA dose was adjusted to 25 mg/m^2^. Coagulopathy was treated with fresh-frozen plasma and apheresis platelets with the objective of maintaining platelet counts above 20G/l, prothrombin time >50%, and fibrinogen >1 g/l. Heparin was not used. Prednisone (0.5 mg/kg/d, days 1–15) was added if WBC >10G/l to prevent differentiation syndrome (DS). Diagnosis of DS, in agreement with previous experiences, was based on the presence of at least three of the following clinical signs and/or symptoms in the absence of alternative explanations: fever, weight gain, respiratory distress, pulmonary infiltrates, pleural or pericardial effusion, hypotension, and renal failure. Patients with four or more of the above signs or symptoms or with a life-threatening manifestation (respiratory failure requiring respiratory assistance, or renal failure requiring dialysis) were classified as having severe DS. Death was attributed to DS when it occurred in patients with a severe form without alternative explanation. In the presence of suspected symptoms of DS, ATRA was transiently discontinued and dexamethasone 10 mg bid for 3 days or more was started. Sweet’s syndrome was defined according to criteria defined by von den Driesch with histologic finding of dermal neutrophil infiltration.^10^ The diagnosis of pseudotumor cerebri was made based on the three following criteria: papilledema, elevated intracranial pressure with a normal cerebral constituency, and normal central nervous system imaging studies.^11^ Scrotal ulcerations were considered related to ATRA if there was no evidence for other possible diagnoses such as bacterial or viral infection.^12^

Patients in CR received 3 risk-adapted consolidation courses.^13^ Intermediate- and high-risk patients received ATRA (45 mg/m^2^ per day for 15 days) combined with idarubicin 12 mg/m^2^ per day for 2 consecutive days in the third course. Patients who were tested negative for PML/RARA at the end of consolidation were started on maintenance therapy with oral mercaptopurine (50 mg/m^2^ per day), oral methotrexate (15 mg/m^2^ per week), and oral ATRA (45 mg/m^2^ per day for 15 days every 3 months) during 2 years. Prognostic factors of the occurrence of DS were analysed with Pearson test for dichotomous variables, and a logistic regression technique for multivariate analysis. P values of 0.05 were considered to be significant. CIR, event free and overall survival were estimated by the Kaplan–Meier method.

## Results:

Between August 2004 and December 2010, 51 patients with APL genetically confirmed by t(15;17) and/or PML/RARA were diagnosed in the Department of Hematology of Aziza Othmana University Hospital, Tunis, Tunisia. One patient died from CNS bleeding before treatment and 50 patients received induction therapy according to the PETHEMA LPA99 trial. Median time from the first symptom to confirmed diagnosis was 15 days (range, 2–90). The main initial clinical symptoms consisted of bleeding (66%), infection (21%), anemia (10%) and deep venous thrombosis (2%). DIC was confirmed biologically in 70% of patients. Main clinical and biological characteristics of the patients are shown in Table 1.

## Induction therapy:

### Hematological and molecular responses

Of the 50 evaluable patients, 43 achieved CR (86%). The remaining seven patients had early death: four caused by DS and three died from CNS hemorrhage. By multivariate analysis female gender (P=0.045), baseline WBC > 10 G/L (P=0.041) and serum creatinine > 1.4mg/dl (P=0.021) were associated with lower CR rate. At the end of induction, 33 (78.5 %) of the 42 patients tested molecularly for PML/RARA were positive.

### Differentiation syndrome and other ATRA-related complications

DS was observed in 16 patients (32%) after a median of 15 days from treatment onset (range, 2-29) and median WBC (at the onset of DS) of 14.7 G/L (range, 0.7–82.7). Clinical symptoms of DS are summarised in table 2.

A hypertensive peak was observed 24 to 96 hours developed DS, of whom only one had a history of (median 36) before DS in 7 (43.7 %) of the pts who systemic hypertension. Systolic and diastolic blood pressure values varied from 160 to 260 mmHg and 95 to 110 mmHg, respectively. DS was severe in 11 cases, moderate in 4, and fatal in four cases. By univariate analysis, age ≥ 40 (P=0.034), BMI ≥ 30 (P=0.009), baseline WBC ≥ 10G/l (p=0.034), serum creatinine >1.4mg (p<0.001) and absence of cytogenetic abnormalities in addition to t(15;17) (p=0.009) were associated with DS. The last factor was not statistically related to other DS predictive factors, but was significantly associated with a patient age greater than 20 years (P=0.019). A hypertensive peak prior to DS was seen in 43.7% of the patients who developed DS compared to 11.7% of those without DS (P=0.011). Occurrence of a hypertensive peak was independent from the use of steroid prophylaxis (P=0.45), but was significantly associated with high BMI (P=0.003). By multivariate analysis, BMI remained an independent predictor of DS (P=0.01).

Other ATRA-related complications observed were: scrotal ulcerations in 4 patients, perineal ulceration in 1 patient, pseudotumor cerebri in 1 patient (aged 32 years), spleen infarction in 1 patient, and Sweet’s syndrome in 2 patients.

### Infectious complications

During the induction phase, hematologic recovery from neutropenia was observed at a median of 30 days (range, d19–d46) from start of chemotherapy. Neutropenic febrile episodes were microbiologically and clinically documented in 25% and 18% of cases, respectively.

Median duration of neutropenia (ANC< 0.5 G/L) during the 1^st^, 2^nd^, and 3^rd^ cycles was 11.5 days (range, 5–26), 13 days (range 8–36) and 13 days (range, 2–34) respectively. Febrile neutropenia occurred in 38%, 67.5% and 48% during the three consolidation courses, respectively.

## Molecular response:

Forty two patients were assessed for molecular response after induction therapy and at the end of consolidation therapy. 33 (78.5%) and 42 (100%) were PML/RARA −ve respectively after induction and consolidation.

## Maintenance treatment:

All 42 patients alive after completing consolidation therapy proceeded to maintenance therapy. Major side effects observed during this phase were headaches and skin dryness during ATRA therapy.

## Outcome:

**Figure 1.**
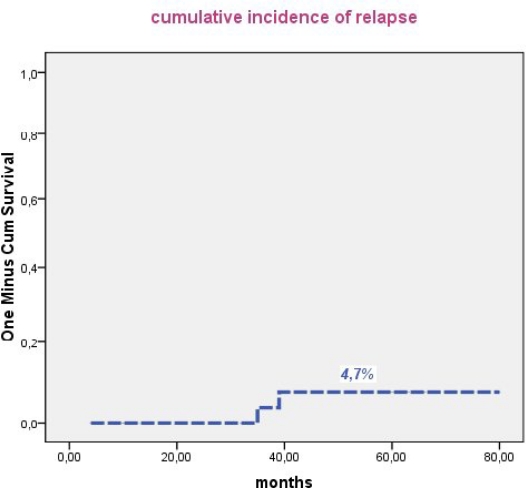
Five year cumulative incidence of relapse

**Figure 2.**
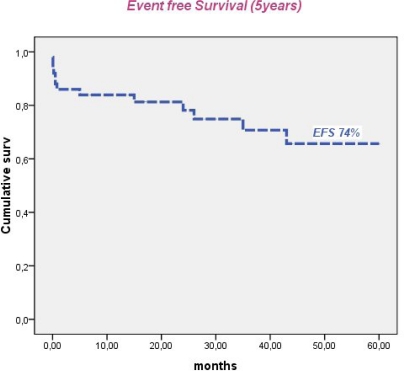
Five year event free survival

**Figure 3.**
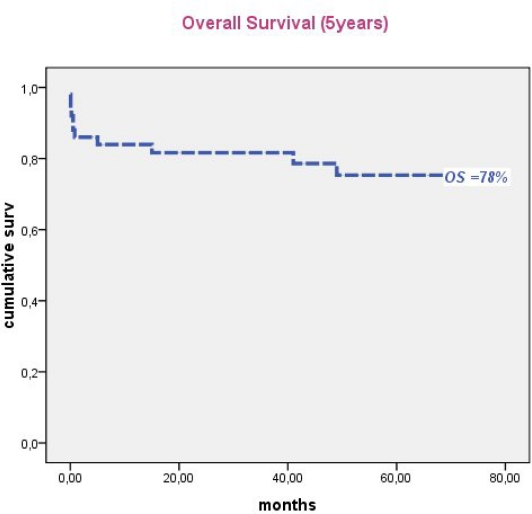
Five year overall survival

Median follow-up of this study was 50 months. Forty patients were alive in continuous complete remission. Two patients, aged 16 and 8 years, died in CR from septic shock during the third consolidation course and from cardiac failure, after 5 and 15 months, respectively. Two relapses occurred in 2 patients (13 and 30 years) of the intermediate risk group after 35 and 36 months of CR, respectively. The first patient (13 yr) was salvaged with two cycles of arsenic trioxide (ATO 0.15 mg/kg i.v. for 25 days) and ATRA (25 mg/m^2^ bid until CR). This patient died from a subsequent relapse 47 months from diagnosis. The second patient was salvaged with the LPA99 protocol and then received autologous stem cell transplantation in negative molecular status. He was 6 months from ASCT with negative PML/RARA. Two patients (41 and 27 years) developed secondary myelodysplastic syndrome with monosomy 7, 25 and 22 months after achieving CR, respectively. The first patient declined bone marrow transplantation and died 47 months from APL diagnosis. The second patient failed FLAG-ida as induction chemotherapy and was still alive in refractory AML. Five year cumulative incidence of relapse, event free survival and overall survival were 4.7% (Figure 1), 74% (Figure 2) and 78% (Figure 3), respectively.

## Discussion:

Our results show that trials combining ATRA and anthracycline based chemotherapy (with or without AraC) designed in industrialized countries can be used at full dose in a country with more limited resources like Tunisia, with CR rates greater than 80% (82% with APL93 trial, and 86% with LPA99 trial).^8^,^9^ As in previous experiences, all failures to achieve CR were due to early death. Our 14% early death rate was however somewhat higher than in recent large industrialized country experiences, probably due in part to the higher proportion of Sanz’s high score (35%) than in most series, and also to Tunisia’s socio economic status, as median interval from first symptoms to diagnosis was 15 days.^14^ Early death in our study was due to hemorrhage (43%) and DS (57%) compared to 58% for hemorrhage, 31% for infection, and 10% for DS in the Spanish experience.^14^ The rate of early death did not decrease in LPA99 study, despite prophylactic use of prednisone (to prevent DS) and tranexamic acid (to improve coagulopathy). Renal dysfunction (creatinine>1.4mg/dl) and high WBC (>10G/L) were the only independent predictors of early death in the PETHEMA study.^14^ Park et al. in an epidemiologic study of the rate of early death in US population-based datasets of all newly diagnosed patients with APL, suggested that the early death rate had changed only modestly since 1992 (22.7% in 1992–1996, and 18.1% in 2002–2007), and was significantly higher than what is reported in contemporary clinical trials.^15^

The high incidence of DS in our experience could probably be explained by the high proportion (35%) of HR patients in our study, and more hypothetically by ethnic differences with other populations. We found higher baseline WBC and increased serum creatinine to be predictors of DS,^16^ as in Spanish studies. We also confirmed the correlation between high BMI ≥ 30 and occurrence of DS, which we had found in a preliminary report with smaller patient numbers.^17^ We then suggested as a possible explanation to this link the fact that leptin receptors, absent from normal promyelocytes are present on APL cells. Leptin secretion by bone marrow adipocytes in the vicinity of leukemic cells could play a major role in the proliferation and survival of APL cells leading to high risk of developing DS.^18^,^19^

We noted hypertensive peak before DS in 43.7% of patients who developed this complication compared to 11.7% of those without DS (P=0.011). Occurrence of a hypertensive peak was independent of the use of steroid prophylaxis, but was significantly associated with high BMI. Increased leptinemia contributes to increased sympathic activity and the development of obesity-related hypotension. Leptin also increases the generation of reactive oxygen species (ROS) in endothelial cells, stimulates secretion of proinflammotory cytokines such as TNF-b and IL-6, both of which are promoters of hypertension and atherosclerosis,^20^ possibly explaining the relationshsip between hypertensive peaks in obese patients prior to the onset of DS. This finding needs to be prospectively validated in a larger series. We also found that patients without cytogenetic abnormalities in addition to t(15,17) had increased risk to develop DS, although there is no clear explanation to his relationship. Four patients, who required ventilation (3 cases) and renal dialysis (1 patient) died from severe DS.

In our study, two patients developed secondary MDS with monosomy 7, a relatively high incidence (4.7%) compared to what has been previously reported.^21^,^22^,^23^

In conclusion despite more limited resources than in industrialized countries, and non availability of arsenic derivatives, our results are quite acceptable. The CR rate we observed is still somewhat inferior to what is reported in international trials. However our 5 year EFS and OS of 74% and 78% are encouraging and suggest that there is no need to adapt protocols by reducing dose-intensity in trials in developing countries.

## Figures and Tables

**Table 1.** Baseline characteristics of the study population

**Age, median, (range)**	30 (4–71)
< 20 y, n (%)	15 (29.4)
≥ 20 y, n (%)	36 (70.6)
**Gender, n (%)**	
Male	20 (39.2)
Female	31 (60.8)
**Baseline WBC, G/l, median (range)**	4.4 (0.6–123)
< 10, n (%)	33 (65)
≥ 10, n (%)	18 (35)
**Platelet count, G/l, median (range)**	17 (1–70)
< 40, n (%)	46 (80)
≥ 40, n (%)	5 (10)
**Hemoglobin, g/L, median, range**	76 (25–135)
**Sanz Score,n (%)**	
Low	2 (4)
Intermediate	31 (61)
High	18 (35)
**Morphology subtype, n (%)**	
Hypergranular	38 (75)
Microgranular	12 (23)
Basophilic	1 (2)
**Immunophenotyping, n (%)**	
CD33+	48 (94.1)
CD13+	49 (96)
CD117+	29 (56.8)
CD34−	45 (80.3)
HLADR−	47 (92.1)
CD2+	9 (17.6)
CD56+	4 (7.8)
**Karyotype, n (%)**	
t(15,17) +ve	43
t(15,17) −ve*	8
t(15,17) with additional cytogenetic abnormalities	17
**Molecular analysis**	
PML/RARA, n (+ve, n)	50 (47)
Isoforms, n (%)	38
Bcr 1	25 (65)
Bcr 2	1 (3)
Bcr 3	12 (32)

* 3 culture failure and 5 normal karyotype

**Table 2:** Clinical symptoms observed in the 16 patients who developed DS.

Symptoms of DS	n (%)
Fever	11 (68.6)
Dyspnea	15 (93)
Weight gain	8 (50)
Pulmonary infiltrates	9 (56)
Pleural/Pericardial effusion	6 (37.5)
Hypotension	3 (18.7)
Renal failure	11 (68.6)
